# Arboreal Ant Colonies as ‘Hot-Points’ of Cryptic Diversity for Myrmecophiles: The Weaver Ant *Camponotus* sp. aff. *textor* and Its Interaction Network with Its Associates

**DOI:** 10.1371/journal.pone.0100155

**Published:** 2014-06-18

**Authors:** Gabriela Pérez-Lachaud, Jean-Paul Lachaud

**Affiliations:** 1 Departamento Conservación de la Biodiversidad, El Colegio de la Frontera Sur, Chetumal, Quintana Roo, Mexico; 2 Centre de Recherches sur la Cognition Animale, CNRS-UMR 5169, Université de Toulouse UPS, Toulouse, France; University of Sussex, United Kingdom

## Abstract

**Introduction:**

Systematic surveys of macrofaunal diversity within ant colonies are lacking, particularly for ants nesting in microhabitats that are difficult to sample. Species associated with ants are generally small and rarely collected organisms, which makes them more likely to be unnoticed. We assumed that this tendency is greater for arthropod communities in microhabitats with low accessibility, such as those found in the nests of arboreal ants that may constitute a source of cryptic biodiversity.

**Materials and Methods:**

We investigated the invertebrate diversity associated with an undescribed, but already threatened, Neotropical *Camponotus* weaver ant. As most of the common sampling methods used in studies of ant diversity are not suited for evaluating myrmecophile diversity within ant nests, we evaluated the macrofauna within ant nests through exhaustive colony sampling of three nests and examination of more than 80,000 individuals.

**Results:**

We identified invertebrates from three classes belonging to 18 taxa, some of which were new to science, and recorded the first instance of the co-occurrence of two brood parasitoid wasp families attacking the same ant host colony. This diversity of ant associates corresponded to a highly complex interaction network. Agonistic interactions prevailed, but the prevalence of myrmecophiles was remarkably low.

**Conclusions:**

Our data support the hypothesis of the evolution of low virulence in a variety of symbionts associated with large insect societies. Because most myrmecophiles found in this work are rare, strictly specific, and exhibit highly specialized biology, the risk of extinction for these hitherto unknown invertebrates and their natural enemies is high. The cryptic, far unappreciated diversity within arboreal ant nests in areas at high risk of habitat loss qualifies these nests as ‘hot-points’ of biodiversity that urgently require special attention as a component of conservation and management programs.

## Introduction

The environmentally buffered nests of most ant species are relatively stable microhabitats in both time and space where resources are readily available [1], [2]. They also confer a certain degree of protection. Thus, ant colonies constitute attractive targets for a wide range of parasites and other symbionts, generally termed myrmecophiles, which are common and can present an impressive diversity. The interactions that myrmecophilous organisms establish with their ant hosts may be facultative or obligatory, direct or indirect (through parasitism of ant guests or prey within the host colony), and range from mutualism to predation or parasitism [1], [3]–[5].

It has recently been pointed out [6] that global conservation priorities focused on vertebrates do not safely cover ants and, most probably, other lesser-known invertebrates. Systematic surveys of macro- and microfaunal diversity within ant colonies are lacking [2], [7], [8], especially in the Neotropics. Ant nests can harbor an impressive and most often unknown biodiversity. For example, more than 500 animal species are known to be associated with the army ant *Eciton burchellii* (Westwood), including ca. 300 arthropods, most of which are awaiting description [9]. The species associated with ants are generally small and rarely collected organisms (as exemplified by numerous parasitic wasps [8], [10]), which makes them more likely to be unnoticed. Small species with narrow host ranges and narrow distributions are known to receive less attention from collectors and taxonomists and to represent the largest proportion of undescribed [11], [12] and missing species [13]. It can reasonably be assumed that this tendency is greater for arthropod communities in microhabitats with low accessibility, such as those found in the canopy [12], and more specifically in the nests of arboreal ants.

In the present study, we surveyed the invertebrates associated with *Camponotus* (*Myrmobrachys*) sp. aff. *textor* Forel, an undescribed Neotropical weaver ant (J.H.C. Delabie, pers. comm., and W.P. MacKay, pers. comm.), by directly evaluating myrmecophile biodiversity within ant nests. Similar to other weaver ants of the genera *Oecophylla* F. Smith, *Polyrhachis* F. Smith and *Camponotus* Mayr [1], *C.* sp. aff. *textor*, which inhabits various species of trees in southern Mexico, builds its nests by sewing leaves together using the silk produced by its larvae. Nests of this species were abundant in the Soconusco region of Chiapas until recently [14], being found on traditional (high-shade) coffee plantations considered to constitute conservation management systems because of the persistence of numerous elements of native plant and animal diversity [15], [16]. However, the coffee plantations in this region are being transformed to low-shade, intensively managed agro-ecosystems [16] and such rapid agricultural intensification combined with biotic homogenization at a large scale may accelerate species losses both locally and at larger spatial scales [17]. Like all of the other ants inhabiting trees, *C.* sp. aff. *textor* is threatened by the rapid fragmentation and conversion of its habitat, although no arboreal ant species has yet been placed on the IUCN red list [18], and we know nothing about either the diversity of organisms associated with these ants or the web of ecological interactions that occur within their nests.

Concerning conservation, it is widely acknowledged that although it is desirable to conserve as many species as possible, we have no knowledge of most of them [11], [19]. Therefore, our intentions are twofold: first, to provide information on the invertebrates associated with this unknown, yet already threatened ant species, and their relationships; and second, to draw attention to ant nests and colonies as a source of cryptic biodiversity. Such ‘hot-points’ of biodiversity that combine endemism and the risk of habitat loss similarly to the biodiversity hotspots defined by Myers [20], though at a more local scale, have been largely neglected. This is particularly the case for arboreal species, most likely because of the rarity of some ant-myrmecophile associations and because the standard sampling methods used to assess ant diversity (fogging, direct sampling, transect walks, baits and traps) generally miss organisms living inside colonies [2]. We also wish to stress the urgent need for such detailed studies as a component of conservation and biodiversity management programs focused on invertebrates.

## Materials and Methods

### Ethics Statement

Colonies were collected from an unprotected private family orchard with no measurable habitat disturbance. They were obtained with appropriate permissions from landowners. Collection and observations comply with the current laws of Mexico in which they were carried out under the official standard NOM-059-SEMARNAT-2010 (Secretaría de Medio Ambiente y Recursos Naturales – Subsecretaría de Gestión para la protección Ambiental).

### Colonies Collection and Data Collection

Insecticide fogging and the use of baited traps are common sampling methods in studies of ant diversity [21]. However, they are not suited for evaluating myrmecophile diversity within ant nests [2]. Here we chose to directly evaluate the macrofauna within ant nests through exhaustive colony sampling and careful examination of every individual within the nest under a stereomicroscope. The laborious nature of our approach and the need to sacrifice all individuals dictated the assessment of reduced colony sample sizes.

Three complete *C.* sp. aff. *textor* nests were collected at the INIFAP Experimental Field Station at Rosario Izapa, Tuxtla Chico municipality, Chiapas, Mexico (14°58′25′′N, 92°09′19′′W, 430 m above sea level), and in an adjacent family-run orchard, at a distance of 300 m, during the dry season (February 2010). One nest was located in an *Inga* sp. tree (Fabaceae) that provides shade for an ancient parcel of coffee plants. The two other nests were located on two mandarin trees (*Citrus reticulata* Blanco, Rutaceae) planted 30 m apart. The nests were collected by placing the entire nest structure in a plastic bag and by cutting off the supporting branch. The nests were then cooled in a refrigerator for 24 hours and subsequently opened, and their contents were preserved in alcohol for later examination. A small sample of the cocoons was placed in a 0.5 l plastic jar with nylon organdy secured over the top to allow parasitoid emergence. All individuals (adults, pupae and larvae) found in each nest were carefully examined under a stereomicroscope and counted. Eggs were not counted but their presence was noted. Adult ants were examined for the presence of male myrmecolacid pupae (Insecta: Strepsiptera: Myrmecolacidae) protruding between the tergites of the abdomen of stylopized specimens, and nematodes (Nematoda: Mermithidae) within the distended abdomens of mermithized specimens. Considering their number (more than 50,000, see Results section), adult ants were mainly visually checked for signs of parasitism by scrutiny of the dorsal surface of their gaster, as generally performed for large colonies (see [22]). For both myrmecolacid strepsipterans [22]–[24] and most mermithid nematodes [25], [26], infection of the ant occurs in the larval stage of the host. Therefore, ant larvae were examined both for the presence of planidia (the first-instar larvae of eucharitids and some other parasitoid wasps and flies) attached to their surfaces and for scars or other external signs of endoparasitism (evidence of dipteran respiratory funnels, changes in color or appearance, unusual pigmentation indicating possible immature parasite eyespots). The larvae of *C.* sp. aff. *textor* pupate within thin silk cocoons, whose interior is clearly visible when preserved in alcohol, allowing the cocoons to be directly examined when backlit. The pupae in cocoons were examined for the presence of immature or adult parasitoids and endoparasites. Whenever any sign of possible parasitism occurrence was suspected, both adult ants and immature stages were systematically dissected; this was the case for 400 adults, 750 pupae and 150 larvae. Any other organisms found inside the nest were also recorded.

Samples of both the ants and myrmecophiles were sent to specialists for identification. Voucher specimens of ants, parasites, parasitoids and other myrmecophiles were deposited in the Arthropod collection of El Colegio de la Frontera Sur at Chetumal, Quintana Roo, Mexico (ECO-CH-AR), in the Natural History Museum of London, England, and in the National Museum of Natural History in Washington, D.C., U.S.A.

## Results

### Composition of the Ant Host Colonies

The collected nests were located on the apical portions of sunny branches, at a height of 3–5 m. Only one nest was present per tree, except for the tree on which nest #3 was collected, where a small abandoned nest was located in its proximity. The nests of mature colonies are oblong or round in shape and can measure up to 40 cm in diameter. Their size is variable and appears to be correlated with colony size.

The three collected colonies were monogynic: only one, large (total length 8.6 mm, n = 3) dealate female, located in the inner chambers of the nest along with plentiful eggs and small larvae, was observed per colony (Table 1). The workers were polymorphic: both major and minor workers were present in all nests, with the major workers being double or more the size of the minor workers. However, the sub-castes were not counted individually, and their relative proportions were not estimated. Four adult males were found within a nest, but no alate females were present in any of the colonies (Table 1). Male and female pupae were present in considerable numbers in the two largest nests. A sex ratio of 0.61 (proportion male) was calculated from the data on sexual pupae from these two nests. Eggs and larvae in different developmental stages were present in all three nests. The average colony size (mean ± SE, n = 3) consisted of 16,734±4,039 workers, 2,656±556 cocoons and 7,380±2,242 larvae (Table 1).

**Table 1:** Composition of the three collected *Camponotus* sp. aff. *textor* colonies and number of parasitized individual hosts.

		Colony # 1	Colony # 2	Colony # 3
	Tree host plant	*Inga* sp.	*Citrus reticulata*	*Citrus reticulata*
	Collection date	02/02/2010	23/02/2010	28/02/2010
Adults	Queens	1	1	1
	Workers	9885	16449	23868
	Alate females	0	0	0
	Males	0	4	0
	Nematode infected workers	0	8	13
	Stylopized workers	0	0	1
	Total parasitized workers	0 (0%)	8 (0.05%)	14 (0.06%)
	Total adults	9886	16462	23883
Immatures	Eggs	++++	++++	++++
	Larvae	3305	7800	11036
	Cocoons (total)	1570	2991	3407
	prepupae	0	654	883
	male pupae	10	285	984
	female pupae	0	411	416
	worker pupae	1543	1614	1088
	Total parasitized cocoons	17 (1.08%)	27 (0.90%)	36 (1.06%)
	Total immatures	4875	10791	14443

### Associated Organisms and Web of Interactions within Host Nests

**Figure 1:**
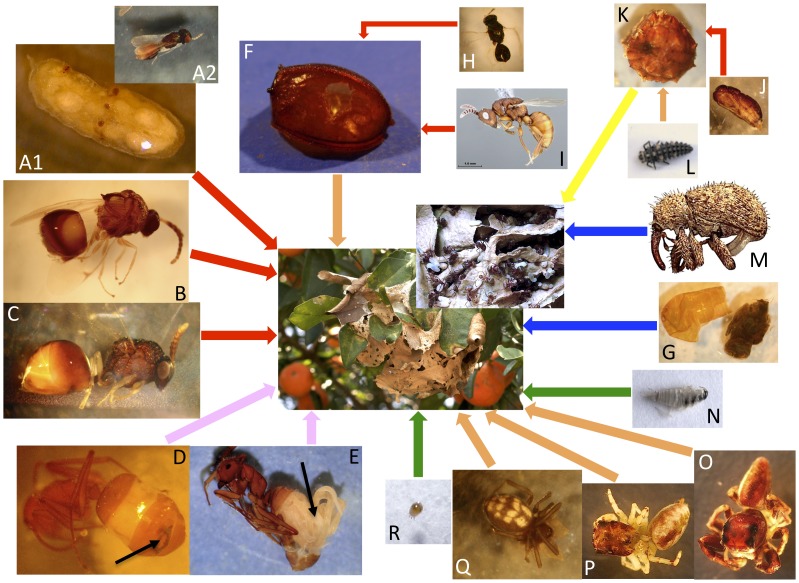
Silk nest of *Camponotus* sp. aff. *textor* (at the center) and its interaction network with its associates. (See Table 2 for the identity of the organisms referred to as A, B, C, …). Red, pink, orange, yellow, green, and blue arrows represent parasitoidism, endoparasitism, predation, mutualism, scavenging and unknown relationship, respectively. The black arrows in pictures D and E indicate the insertion of a myrmecolacid Strepsiptera and a mermithid Nematoda, repectively, in the gaster of an adult *Camponotus* worker. The diversity of the community of invertebrates associated with *C.* sp. aff. *textor* corresponds to an equally diverse array of relationships with the host.

Globally, the number of *C.* sp. aff. *textor* adults and immature stages parasitized was quite low (Table 1). Among the examined colonies, only 1% of the cocoons had been attacked by parasitoid wasps, and only 0.04% of adults (workers only) had been attacked by nematode or strepsipteran parasites. Larvae did not show any sign of parasite attack. The dissection of 150 larvae did not yield immature stages of strepsipterans nor nematodes and, in a dozen cases, we only noticed an accumulation of a whitish, dense substance that might be the expression of the host immune defense mechanism, possibly denoting the failure of a parasitic attack. Despite this low prevalence of parasitism, invertebrates belonging to at least 18 taxa from three classes (Table 2) were found inside the nests of *C.* sp. aff. *textor*, and were observed to be involved in some sort of myrmecophilic relationship with their host (Fig. 1).

**Table 2:** Invertebrate myrmecophiles found within the nests of the weaver ant *Camponotus* sp. ca. *textor* and nature of the relationship with the host.

	Species	Nature of the relationship	Colony #1 on *Inga* sp. 02/02/2010	Colony #2 on *C. reticulate* 23/02/2010	Colony #3 on *C. reticulate* 28/02/2010
**Parasitoids**					
Insecta, Hymenoptera, Eulophidae	*Horismenus myrmecophagus* [A1, A2]	gregarious endoparasitoid of ant larvae/pupae	17 parasitized ant pupae	17 parasitized ant pupae	30 parasitized ant pupae
Insecta, Hymenoptera, Eucharitidae	*Obeza* sp. [B]	solitary ectoparasitoid of ant larvae/pupae	0	0	6 (1 ♀, 1 ♂, 1 ♀ P, 3 L)
	*Pseudochalcura americana* [C]	solitary ectoparasitoid of ant larvae/pupae	0	10 (1 ♀, 1 ♀ and 3 ♂ P, 5 L)	0
**Endoparasites**					
Insecta, Strepsiptera, Myrmecolacidae	*Caenocholax* sp. [D]	endoparasite of larval, pupal and adult ants	0	0	1 (♂ P)
Nematoda, Mermithidae	Unidentified [E]	likely endoparasite of larval, pupal and adult ants	0	8 (immatures)	13 (immatures)
**Other myrmecophiles**					
Insecta, Diptera,Syrphidae, Microdontinae	Unidentified [F]	predator of ant brood	0	0	1 parasitized larva
Insecta, Diptera	Unidentified [G]	unknown	0	0	1 puparium
Insecta, Hymenoptera, Eulophidae	*Horismenus microdonophagus* [H]	*gregarious endoparasitoid of microdontine larvae	–	–	1 batch (76 ♀, 6 ♂)
Insecta, Hymenoptera, Eurytomidae	*Camponotophilus delvarei* [I]	*gregarious ectoparasitoid of microdontine larvae	1 adult (1 ♀)	2 adults (2 ♀)	3 adults (3 ♀)
Insecta, Hymenoptera	Unidentified [J]	*endoparasitoid of *Cryptostigma* sp.	present	–	–
Insecta, Hemiptera, Coccoidea	*Cryptostigma* sp. [K]	trophobiont	present (at least 70)	absent	absent
Insecta, Coleoptera, Coccinellidae	Unidentified [L]	*predator of *Cryptostigma* sp. (?)	0	0	1 larva
Insecta, Coleoptera, Curculionidae	*Melexerus hispidus* [M]	unknown	1 adult	0	0
Insecta, Orthoptera, Blattellidae	Unidentified [N]	scavenger	0	1 nymph	0
Arachnida, Araneae,Salticidae	Unidentified sp. 1 [O]	predator of ant adults or brood	0	1 adult (1 ♀)	0
	Unidentified sp. 2 [P]	predator of ant adults or brood	0	1 adult (1 ♀)	0
Arachnida, Araneae, (other)	Unidentified [Q]	predator of ant adults or brood	0	1 adult (1 ♀)	0
Arachnida, Acari	Unidentified [R]	scavenger	1 adult	0	0

Column #2: the capitals between brackets refer to the identity of the myrmecophiles in Figure 1.

Columns # 4–6: the values correspond to the number of myrmecophile individuals (or the number of parasitized hosts in the case of *Horismenus myrmecophagus*). P = pupae, L = larvae.

*Indirect association within the ant nest, through another guest.

Three parasitoid wasp species, belonging to two families, attacked the larvae of *C.* sp. aff. *textor* (Table 2) but emerged from cocoons (koinobiont development): the recently described gregarious endoparasitoid, *Horismenus myrmecophagus* Hansson, Lachaud & Pérez-Lachaud (Eulophidae: Entedoninae [7]), identified only from females, and the solitary ectoparasitoids *Obeza* sp. and *Pseudochalcura americana* (Howard) (Eucharitidae: Eucharitinae), for which both sexes were present. Individuals of both eucharitids and the eulophid co-occurred within the same nest.

Workers were attacked by two endoparasites from two invertebrate classes: one myrmecolacid Strepsiptera (a single worker was parasitized by a male *Caenocholax* sp. pupa) and, numerous (22 cases in total) mermithid nematodes. Each nematode infected worker (only workers were parasitized) harbored a single nematode occupying the entire distended gaster (Fig. 1). Only immature stages of the nematodes were obtained, which precluded their identification.

Several species of potential ant predators were registered, though in very low numbers. These predators included a single larva of an unidentified microdontine fly (Diptera: Syrphidae), which is a group that is known to predate on ant host brood [27] (but see [28]), and three different species of spiders (including two Salticidae species), presumably predating the host larvae. Scavengers were also rare, and we only found one species of mite (Arachnida: Acari) and one cockroach nymph (Orthoptera: Blatellidae).

Various myrmecophiles found within the nests of *C.* sp. aff. *textor* were also parasitized by different parasitoids or were attacked by predators within the nest. The unidentified microdontine larva mentioned above was parasitized by another gregarious eulophid wasp, *Horismenus microdonophagus* Hansson, Lachaud & Pérez-Lachaud (Entedoninae), which was recently described [7]. One ladybird larva (Coleoptera: Coccinellidae), found in colony #3, was most likely a predator of a hemipteran exploited by *C.* sp. aff. *textor* as hemipteran trophobionts can occasionally be found within ant host nests (Table 2). For example, nest #1 was constructed around an *Inga* sp. branch that exhibited more than 70 *Cryptostigma* sp. soft scales (Hemiptera: Coccidae) of variable sizes. Some of these scales were themselves parasitized by an unidentified wasp parasitoid, possibly a Eulophidae (no adults were available for identification purposes). Other myrmecophiles found inside the weaver ant nests included six gravid females of the recently described eurytomid wasp *Camponotophilus delvarei* Gates [29] which is a gregarious parasitoid of microdontine syrphid larvae that parasitizes its host within the protective walls of the ant nest [30], in addition to the puparium of an unidentified fly and a single specimen of the enigmatic anthonomine weevil *Melexerus hispidus* Burke (Coleoptera: Curculionidae), whose relationship with the host remains unknown.

## Discussion

The invertebrates that associate with ants in various ways include a great diversity of insects (Diptera, Hymenoptera, Coleoptera, Hemiptera, Orthoptera, Thysanoptera and Strepsiptera), and also numerous organisms from other invertebrate classes (arachnids, malacostracans, gastropods, nematodes, trematodes, cestodes, centipedes and millipedes) [1], [3]–[5]. However the lack of knowledge regarding most organisms associated with ants is a general problem, as data on the biology of these species are frequently missing, the entire life cycle is unknown for most of them and, in numerous cases, their myrmecophily is only inferred from indirect, unreliable evidence [31]. According to Hölldobler & Wilson [1], the greatest diversity of species of myrmecophiles is to be found within host species that form exceptionally large mature colonies, such as some ecitonine ants [9]. By contrast, very few symbionts are expected to be found in nests of species with the smallest mature colony sizes (Dacetinae, Leptothoracinae, Amblyoponinae, Ectatomminae, Heteroponerinae, Paraponerinae, Ponerinae, and Proceratiinae) [1], [2]. Nevertheless, even ant species from these subfamilies can be associated with a considerable number of different symbionts involved in an intricate web of interactions. For example, colonies of the ectatommine ant *Ectatomma tuberculatum* (Olivier) have been observed to harbor at least three different species of eucharitid wasps, a nematode, acari, a gastropod, a small diplopod (Merocheta), a lepismatid or nicoletiid thysanuran, a worm infesting refuse dumps and a myrmecophilous beetle (Histeridae) [32], [33].

In the medium sized colonies of *C.* sp. aff. *textor*, despite the small number of colonies sampled in the present study and the limited number of individuals dissected, the community of associated invertebrates was found to be highly diversified, including specimens of at least 18 taxa from three classes (Insecta, Arachnida, and Nematoda). One genus and three species of parasitic wasps that are new to science have been described elsewhere from the material collected in this study [7], [29], and various other myrmecophiles await identification or description, particularly the relatively frequent mermithid nematode species reported here, as most mermithids attacking ants are known to be host-specific [26].

Various invertebrates, particularly lycaenid butterflies and spiders, have previously been reported to be associated with *Oecophylla* weaver ants in the old world (see [34] for a review) and a few species have been reported to be associated with *Polyrhachis* [35]. However, nothing was known about invertebrate myrmecophiles living within the nests of *Camponotus* or any other weaver ant in the new world prior to our work. Trophobionts have been reported for different species of *Camponotus* (*Karavaievia*) [36], *Oecophylla* [37], [38] and *Polyrhachis* [35], [39] in Africa, Malaysia, Asia and Australia. Mutualistic interactions between Neotropical *Camponotus* ants and soft scale insects of the genus *Cryptostigma* Ferris have only been reported previously for *C. longipilis* Emery, *C. mirabilis* Emery and *C.* (*Pseudocolobopsis*) sp. inhabiting bamboo plants in Peru [40] and for *C. novogranadensis* Mayr within a termitary of *Amitermes excellens* (Silvestri) in British Guiana [41]. Only three predatory salticid spiders have been identified from the nests of *O. smaragdina* (Fabricius) and *O. longinoda* (Latreille) or their proximity: *Cosmophasis bitaeniata* (Keyserling) [42] and *Myrmarachne plataleoides* (Pickard-Cambridge) [43] in Australia and Asia, and *M. foenisex* Simon in Africa [44]. The two unidentified salticid species and the other unidentified Araneae reported here from Mexico are the first spiders to be found within *Camponotus* weaver ant nests. Some eucharitids have previously been reared from weaver ant pupae from the old world (*Stilbula polyrhachicida* Wheeler & Wheeler from *P. dives* F. Smith [45], *Rhipipalloidea mira* Girault from *P. femorata* F. Smith and *R. madangensis* Maeyama, Machida & Terayama from *Camponotus* (*Tanaemyrmex*) sp. [46]), and some species of *Smicromorpha* Girault (Chalcididae) are parasitic on larvae of *O. smaragdina* [47]. However, until the present work, the eucharitid genus *Obeza* Heraty had only been found in association with the carpenter ant *Camponotus floridanus* (Buckley) in Florida [48] and the host of *Pseudochalcura americana* was hitherto unknown. Of particular note, both Eucharitidae and Eulophidae were found parasitizing larvae from the same nest. The co-occurrence of two eucharitid species attacking the same ant colony has been observed previously [32], but the co-occurrence of two different parasitic wasp families attacking the brood of a single ant nest has not been reported elsewhere. Although various Palearctic *Camponotus* species have been found to serve as hosts of mermithid nematodes [26], such findings are rare for Neotropical *Camponotus* [25] and consist of only a single unidentified species attacking *Camponotus atriceps* (F. Smith) in Mexico [49] and another unidentified species attacking *C. punctulatus minutior* Forel in Argentina [50]. In our samples, attacks by nematodes were common and more prevalent than parasitism, for example, by strepsipterans, although this finding could be an artifact because mermithized workers with enlarged abdomens are much more conspicuous than stylopized workers and more readily noticed. Among nearly 50,000 adult workers examined, only one was stylopized, and thought 400 adults with an unusual appearance were dissected, no definitive conclusions could be drawn from these specimens. No strepsipteran parasitism of ant pupae or larvae was either noticed, even though dissections of immature stages reported here (750 pupae and 150 larvae) largely outnumbered those usually performed to check for strepsipteran parasitism of ants (see [22], [23]) (but see [51] for parasitism of *Polistes* wasps). However, considering the small percentage of individuals dissected, it is very likely that we underestimated strepsipteran prevalence. Due to high prevalence in infection of *Dolichoderus bispinosus* males [22], it has been suggested that ant castes could be differentially parasitized, but this likely does not apply for *C.* sp. aff. *textor* because none of the 4 alate males we collected were stylopized and none of the numerous male and female pupae present in the nests did present any sign of parasitism. Nevertheless, because strepsipterans are infectious organisms and as such must maintain high enough numbers to transmit, it is likely that sampling *C.* sp. aff. *textor* nests at another season would yield more significant results. Strikingly, myrmecophilous beetles were very rare within the examined nests of *C.* sp. ca. *textor* (a single coccinellid larvae and a single curculionid adult), whereas they are present in great numbers with high diversity in the colonies of many other ants [3], [9]. The absence of any refuse within the nests of this ant species (*C.* sp. aff. *textor* covers food waste and other remains with silk) may account for the absence of scavenging beetles.

The life histories of some of the myrmecophiles reported here (e.g., the three spider species, the curculionid beetle and the dipterans) clearly deserve further attention to elucidate the exact nature of their relationships with the ants. The results of the present study draw attention to the complexity of the interactions occurring within the colonies of *C.* sp. aff. *textor*. The diversity of the invertebrate associates of this ant species corresponded to an equally diverse array of relationships with the host (Fig. 1): mutualism (between the ants and their coccid trophobionts), relatively passive parasitism (by scavengers), aggressive parasitism of both adult ants and brood (by parasitoid wasps and endoparasitic nematodes and strepsipterans), predation (by spiders and syrphids) and various indirect relationships involving parasitism (by other parasitoid wasps) or predation (by a ladybird larva) of various myrmecophiles (coccids, syrphid flies) were involved simultaneously in a highly complex interaction network. The use of molecular diagnostic techniques for examining host/parasitoid and prey/predator associations through analyses of the gut contents of adult parasitoids and predators (see [52]) would certainly help to resolve some of the missing links in this interaction network.

Hughes et al. [2] hypothesized that in the nests of large, relatively long-lived insect societies, the large number of workers and coordinated hygienic defense would likely impose selective pressure on parasites to reduce their virulence. It was also argued that under such conditions, social insects with large colonies will likely accumulate a much higher load of diverse, low-cost parasites over their evolutionary history than similar species with small colonies or solitary sister groups. Our results showed that whereas various myrmecophiles were associated with *C.* sp. aff. *textor*, although the figures reported here might not reflect the actual parasitism rate, the global prevalence of parasitism was remarkably low. Agonistic interactions with this ant prevailed, considering the co-occurrence of three parasitoids, two endoparasites and four predators inside the nests, but very few pupae and adult workers were actually parasitized, and no larva (out of 22,141) showed any apparent evidence of parasite attack. These results are almost certainly an underestimate because of the difficulty of identifying parasitized larvae in early stages of attack by endoparasites and because stylopized and mermithized workers were detected only when the parasites were fully grown and the adults were ready to emerge from the host. Nevertheless, the other invertebrates associated with *C.* sp. aff. *textor* were also found in very low numbers. Our findings therefore support the hypothesis of Hughes et al. [2] that the virulence of symbionts or myrmecophiles associated with large, long-lived insect colonies would be modulated over evolutionary time, while their diversity within these colonies would increase. The very low prevalence of most of these myrmecophiles may also explain why they have been unnoticed until now.

The macro- and microfauna associated with ants and other social insects are likely to contribute significantly to both ecosystem biodiversity and biomass [2], and the urgent need for detailed surveys focusing on their cryptic diversity has recently been stressed on various occasions [4], [5]. Natural tropical and Neotropical habitats exhibit exceptionally high invertebrate diversity [19] but are also subject to a high risk of degradation [20], [53]. Despite this state of affairs, performing a global inventory remains a difficult task. For example, systematic, detailed surveys of the macrofauna present within the colonies of Neotropical arboreal ants had not been undertaken prior to this study. Rare species are believed to be more prone to extinction than common species [54], and species from higher trophic levels (e.g., parasitoids, hyperparasitoids and predators) are also particularly threatened. Thus, several species of social parasites of ants involved in highly specific relationships with their hosts are already considered endangered because of their high vulnerability to habitat change [55], [56], and this situation is even more obvious for the specific parasitoids of these parasites [30]. Recent evidence has suggested that, similar to many other arboreal ant species found in ‘hotspots of discovery’ [53], *C.* sp. aff. *textor* and its associated fauna are about to fall into the threatened category because of the loss of their habitat. Recent surveys conducted in 2011 and 2012 [30] did not detect any silk nests at our study site, where the experimental shaded coffee plantation has now been transformed into a biofuel plantation with no shade trees. Such habitat transformation tends to expand in space and is generally the rule in the southern region of Mexico [16], where this ant species was considered common only a few years ago [14]. As shown by Chase & Knight [57], environmental changes such as habitat degradation can have scale-dependent effects, with proportionately larger effects being observed in more infrequent species. Because the myrmecophiles found in this work are rare and most of them are strictly specific and exhibit highly specialized biology, the risk of extinction for these hitherto unknown arthropods and their natural enemies is high. The number of species on earth has recently been estimated at 8.7 million, 86% of which are yet to be discovered and described [13]. Such species are likely to present small ranges and to be concentrated in hotspots and poorly explored areas [13], [53]. As noted by Krauss et al. [58], “*in present-day fragmented and perturbed landscapes, populations of many species might be on a deterministic path to extinction even without any further habitat loss occurring*”. The cryptic and thus far unappreciated diversity of invertebrates within arboreal ant nests in areas at high risk of habitat loss qualifies these nests as ‘hot-points’ of biodiversity that deserve special attention in the very near future as a component of conservation and biodiversity management programs.
